# Smart bats click twice

**DOI:** 10.7554/eLife.36561

**Published:** 2018-04-13

**Authors:** Manfred Kössl, Julio Hechavarría

**Affiliations:** 1 Institute for Cell Biology and NeuroscienceGoethe UniversityFrankfurt/MainGermany

**Keywords:** superior colliculus, echolocation, free flying, 3D receptive fields, bats, Eptesicus fuscus, Other

## Abstract

The acoustic representation of the outside world in the midbrain of a bat becomes more precise as it uses double clicks to locate closer objects.

**Related research article** Kothari NB, Wohlgemuth MJ, Moss CF. 2018. Dynamic representation of 3D auditory space in the midbrain of the free-flying echolocating bat. *eLife* **7**:e29053. doi: 10.7554/eLife.29053

To navigate their environment, humans and other animals need to be aware of their surroundings and their position relative to other objects. This ability to perceive space is crucial for survival and usually relies on all our senses conveying sensory information to the brain, which computes a model of the outside world that enables the animals to act accordingly. While humans gather acoustic and visual information ‘passively’, some animals have developed sensory systems that actively explore their surroudings.

For example, bats use a process called echolocation to orient themselves in space and to search for food. This involves emitting high-frequency sound waves from their nose or mouth, and then detecting the echoes of these waves after they have been reflected by a nearby object (Figure 1). The time taken for the echo to reach the bat helps it to estimate the distance and direction of the object and to create a direct perception of the depth of the space that surrounds them.

**Figure 1.**
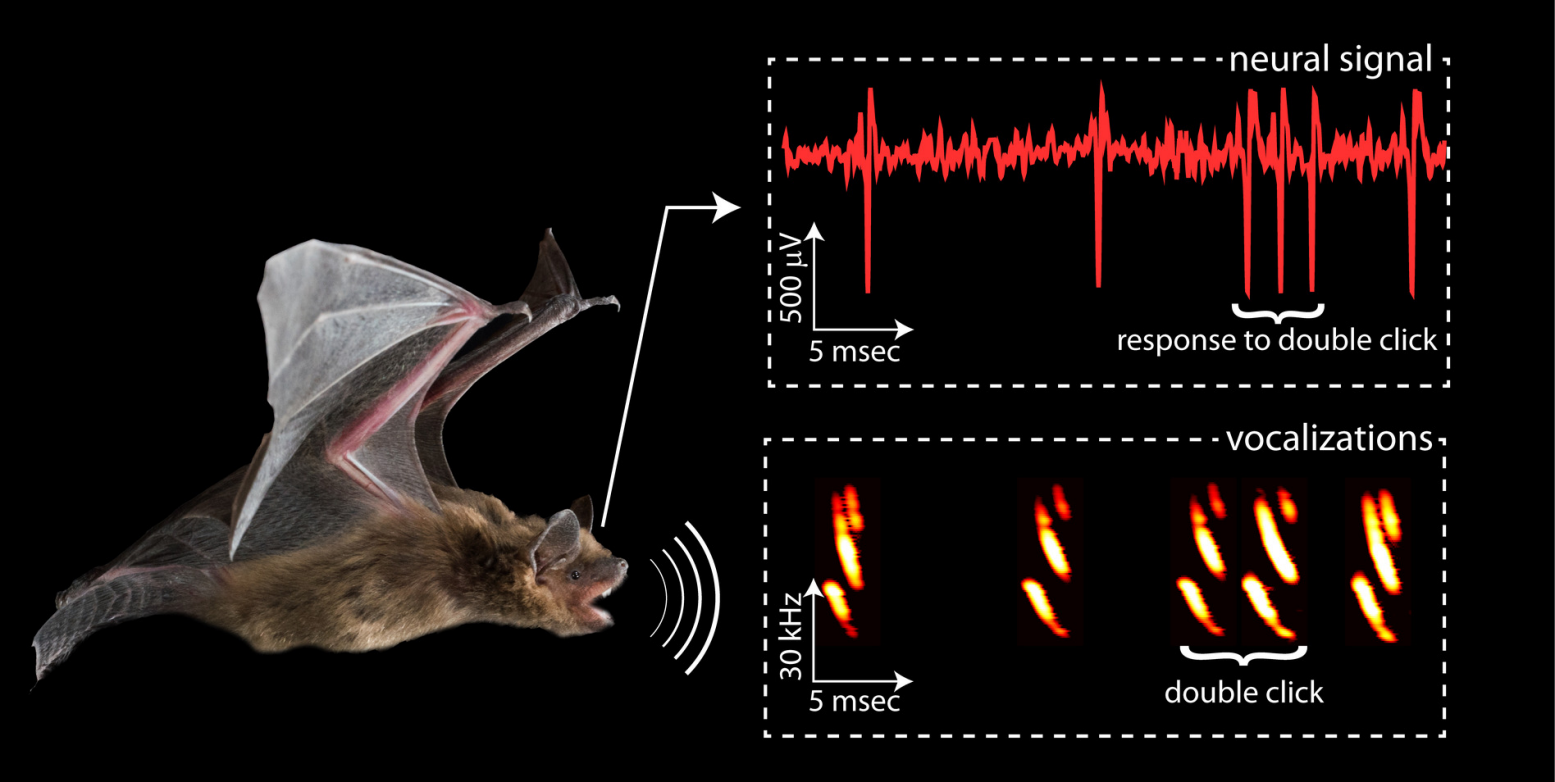
Different approaches to echolocation. Bats use sound waves to see in the dark. Most of the time, big brown bats emit a single 'click' of sound (as shown on the left of the rectangle labelled vocalizations), and a single spike is generated in the brain when the echo of this click is detected (as can be seen on the left of the rectangle labelled neural signal). However, when a bat gets close to an object it emits a double click, which leads to the generation of a more complex signal in the brain (three spikes in this case). Kothari et al. found that during double clicking a group of neurons in the superior colliculus, which is in the midbrain, responded more strongly to close objects. Image credit: Melville Wohlgemuth.

Most previous research in this area has focused on how the brain processes either the direction or the distance when the bats are restrained in some way. Two regions in the brain are thought to be involved in encoding spatial depth: the auditory cortex, and a region in the midbrain called the inferior colliculus (Suga and O'Neill, 1979; Wenstrup and Portfors, 2011). However, when the bats are free to move, the distances and directions of objects change constantly, and relatively little is known about how the brain deals with these changes.

Now, in eLife, Ninad Kothari, Melville Wohlgemuth and Cynthia Moss at Johns Hopkins University report that another region in the midbrain, the superior colliculus, plays an important role in estimating distance (Kothari et al., 2018). The superior colliculus is known to merge visual and auditory information and to direct behavioral responses towards stimuli in the environment. In bats, it is thought to be involved in controlling sensory-guided behaviors that help the animals to orientate themselves by using acoustic signals or adjusting their body or ears towards objects detected by echolocation (Covey et al., 1987; Valentine et al., 2002).

Kothari et al. inserted wireless probes into the midbrain region of bats and recorded the electrical impulses in their brains as they flew in three-dimensional space; the echolocation signals were also recorded. The researchers discovered a group of neurons in the superior colliculus that compute a three-dimensional representation of the space around the bat by processing information about the distance in between objects.

Bats can control their ‘acoustic view’ of the world by changing the temporal structure of their echolocation streams. At closer distances, they often use pairs of echolocation signals that are emitted in short succession, also known as double clicks (Kothari et al., 2014; Figure 1). Kothari et al. show that by using the double clicks to focus on a nearby object, the activity in the brain shifted. The neurons in the superior colliculus fired more frequently when close objects were inspected and registered the distance to them in better detail. This implies that the representation of the spatial surroundings in the midbrain becomes more precise.

The double clicking was also associated with an increase of electrical activity at high frequencies (between about 50 and 140 Hertz) in the midbrain. This 'gamma activity' is known to emerge when groups of neurons are involved in attention tasks (Fries, 2009). For example, gamma activity in the superior colliculus of barn owls increases when the birds focus their attention on specific objects (Sridharan and Knudsen, 2015), which seems to be similar to what happens in bats.

Overall, the work of Kothari et al. offers us a unique view on the bat’s sensory world as it flies through the dark. It shows for the first time that these animals create an acoustic representation of the external world in a region in the brain that specifically controls fast behavioural reactions to objects and obstacles. It also establishes bats as a promising model to study the mechanisms of sensory attention and the interaction between sensory and motors systems.
